# Differentially Altered NMDAR Dependent and Independent Long-Term Potentiation in the CA3 Subfield in a Model of Anti-NMDAR Encephalitis

**DOI:** 10.3389/fnsyn.2018.00026

**Published:** 2018-07-31

**Authors:** Roman Blome, Willi Bach, Xiati Guli, Katrin Porath, Tina Sellmann, Christian G. Bien, Rüdiger Köhling, Timo Kirschstein

**Affiliations:** ^1^Oscar Langendorff Institute of Physiology, University of Rostock, Rostock, Germany; ^2^Epilepsy Center Bethel, Krankenhaus Mara, Bielefeld, Germany

**Keywords:** associational-commissural fibers, mossy fibers, LTP, NMDA receptor, epileptiform afterpotentials

## Abstract

**Purpose**: Autoantibodies against NMDA receptors (NMDAR) in the cerebrospinal fluid (CSF) from anti-NMDAR encephalitis patients have been suggested to be pathogenic since in previous studies using patient CSF, NMDAR-dependent processes such as long-term potentiation (LTP) were compromised. However, autoantibodies may represent a family of antibodies targeted against different epitopes, and CSF may contain further autoantibodies. Here, we tested the specificity of the autoantibody by comparing NMDAR-dependent and NMDAR-independent LTP within the same hippocampal subfield, CA3, using CSF samples from four anti-NMDAR encephalitis patients and three control patients.

**Methods**: We performed a stereotactic injection of patient-derived cell-free CSF with proven presence or absence of NMDAR-antibodies into the rat hippocampus *in vivo*. Hippocampal brain slices were prepared 1–8 days after intrahippocampal injection, and NMDAR-dependent LTP at the associational-commissural (A/C) fiber-CA3 synapse was compared to NMDAR-independent LTP at the mossy fiber (MF)-CA3 synapse.

**Results**: The LTP magnitude at A/C fiber-CA3 synapses in slices from control-CSF-treated animals (168 ± 8% *n* = 54) was significantly higher than LTP in slices from NMDAR-CSF-treated animals (139 ± 9%, *n* = 40; *P* = 0.015), although there was some variation between the individual CSF samples. We found residual LTP in NMDAR-CSF-treated tissue which could be abolished by the NMDAR inhibitor D-AP5. Moreover, the CA3 field excitatory postsynaptic potential (fEPSP) was followed by epileptiform afterpotentials in 5% of slices (4/78) from control-CSF-treated animals, but in 26% of slices (12/46) from NMDAR-CSF-treated animals (*P* = 0.002). Application of the LTP-inducing paradigm increased the proportion of slices with epileptiform afterpotentials, but D-AP5 significantly reduced the occurrence of epileptiform afterpotentials only in NMDAR-CSF-treated, but not in control tissue. At the MF synapse, no significant difference in LTP values of control-CSF and in NMDAR-CSF-treated tissue was observed indicating that NMDAR-independent MF-LTP is intact in NMDAR-CSF-treated tissue.

**Conclusion**: These findings indicate that anti-NMDAR containing CSF impairs LTP at the A/C fiber-CA3 synapse, although there is substantial variation among CSF samples suggesting different epitopes among patient-derived antibodies. The differential inhibition of LTP at this synapse in contrast to the MF-CA3 synapse suggests the specificity and underlines the pathophysiological role of the NMDAR-antibody.

## Introduction

Limbic encephalitis (LE) is commonly associated with impaired hippocampus-dependent memory function; especially when patients harbor autoantibodies against *N*-methyl D-aspartate receptors (NMDARs; Titulaer et al., 2013; Lynch et al., 2018). The most attractive molecular mechanism for information storage in the brain is believed to be long-term potentiation (LTP)—discovered more than four decades ago (Bliss and Lomo, 1973; Bliss and Collingridge, 2013). Thus, NMDAR activation was demonstrated as being a prerequisite for LTP induction and learning evidenced at various types of hippocampal synapses *in vitro* and *in vivo* (Collingridge et al., 1983; Harris et al., 1984, 1986; Morris et al., 1986; Wigström et al., 1986). With respect to pathophysiology of anti-NMDAR encephalitis, the most striking hypothesis is that NMDAR autoantibodies block synaptic LTP and thereby impair memory performance. Indeed, recent reports have demonstrated that both commercial NMDAR antibodies and anti-NMDAR encephalitis patient-derived cerebrospinal fluid (CSF) containing autoantibodies against NMDARs blocked LTP (Zhang et al., 2012; Dupuis et al., 2014; Würdemann et al., 2016). Together, these studies suggest that autoantibodies directed against neuronal surface proteins such as NMDARs, are pathogenic and both necessary and sufficient for memory impairment in LE patients (Linnoila et al., 2014). However, autoantibodies may comprise a family of antibodies targeted against different epitopes within the NMDA receptor. Thus, the specificity of these autoantibodies in a given patient is an unresolved issue, and it is conceivable that the variance of clinical presentation can in part be explained by different specificities of their antibodies.

Most, but not all forms of hippocampal LTP are NMDAR-dependent. In the CA3 area, two distinct afferent pathways converge onto the same pyramidal neurons. On the one hand, associational-commissural (A/C) fibers terminate on distal parts of CA3 apical dendrites displaying a gradient of NMDAR densities towards higher distal expression (Monaghan and Cotman, 1985). In line with this, A/C fiber synapses within the stratum radiatum (s.r.) show typical cooperative Hebbian LTP that requires NMDAR activation (Zalutsky and Nicoll, 1990, 1992; Katsuki et al., 1991). On the other hand, LTP at the mossy fiber (MF) input terminating on the proximal apical dendrites of CA3 pyramidal cells within the stratum lucidum (s.l.) was demonstrated to be specific, but not cooperative and, in addition, was attained under NMDAR inhibition (Harris and Cotman, 1986; Williams and Johnston, 1988).

Hence, the CA3 subfield offers the unique opportunity to compare NMDAR-dependent and independent forms of LTP within the same area. Thus, we asked whether NMDAR-dependent and independent forms of hippocampal LTP in this area may be differentially affected in slices from animals that have undergone stereotactic intrahippocampal injection of anti-NMDAR encephalitis patient-derived CSF containing NMDAR autoantibodies (Würdemann et al., 2016). We thereby addressed the question of specificity of NMDAR-antibodies and compared CSF with NMDAR-antibodies from different patients. The data show significant LTP reduction at A/C fiber-CA3 synapses in NMDAR-antibody-treated animals compared to controls, but substantial variation of LTP suppression among anti-NMDAR encephalitis patients as well as intact LTP at MF-CA3 synapses.

## Materials and Methods

### Stereotactic Surgery With Intrahippocampal CSF Injection

**Figure 1:**
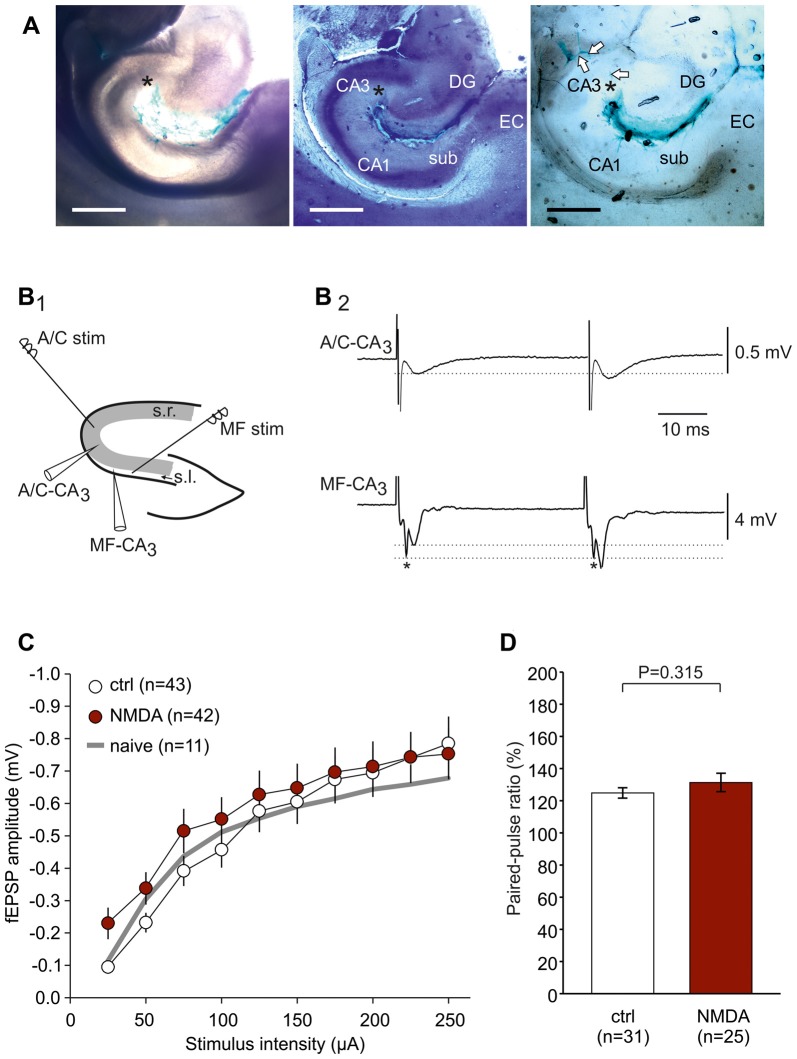
Experimental design to study associational-commissural (A/C) and mossy fiber (MF) input in CA3. **(A)** Microphotographs showing the ink dispersion in the hippocampus 1 h after injection into CA3 stratum radiatum (s.r.; denoted by an asterisk). Magnification 40×. Leftmost panel: native slice (350 μm). Middle panel: air-dried slice stained with toluidine blue and hematoxylin in order to demonstrate hippocampal cell layers. DG, dentate gyrus; sub, subiculum; EC, entorhinal cortex. Rightmost panel: air-dried slice indicating diffusion of ink along the vessels towards CA3 (see arrows). The scale bar indicates 1000 μm. **(B_1_)** Localization of stimulation and recording electrodes in the hippocampus. A/C fiber responses were evoked by stimulation placed in s.r. at the border between CA2 and CA3 and registered within CA3 s.r. MF responses were evoked by stimulation placed in stratum lucidum (s.l.) close to the dentate gyrus and registered within CA3 s.l. **(B_2_)** Typical responses of A/C fiber and MF stimulation showing the characteristic paired-pulse ratios (PPR; indicated by dotted lines). Note that MF responses typically contain a fiber volley (*) which does not express paired-pulse plasticity. **(C)** Input-output (I/O) curves showing no significant difference between control-CSF and NMDAR-CSF slices (*P* = 0.062, two-way-ANOVA). The I/O curve of naive, non-operated animals is indicated by a gray line. **(D)** Paired-pulse ratio did not differ between control-CSF and NMDAR-CSF treated groups (*P* value calculated by using Mann-Whitney U-test).

LE patient derived CSF containing NMDAR-antibodies was stereotactically injected into the hippocampus on both sides *in vivo* as described previously (Würdemann et al., 2016). Briefly, following anesthesia with S-ketamine (100 mg/kg i.p.) and xylazine (15 mg/kg i.p.) female Wistar rats (190–320 g, 60–90 days old) were mounted on a stereotactic frame (Narishige, Tokyo, Japan), and native, cell-free non-diluted CSF (10 steps of 0.5 μl every 2 min, total of 5 μl for each side) was injected using a Hamilton syringe (75 N; Hamilton AG, Bonaduz, Switzerland). This syringe was carefully inserted into the hippocampus with the following coordinates: 5.2 mm posterior, ±4.3 mm lateral, 4.8 mm deep (relative to Bregma). Female rats were chosen since age-dependent changes in scull and brain size are less prominent as in males. The injection site targeting CA3 was confirmed using ink injection (Figure 1A). After the last step of injection, the syringe was left within the injection site for another 2 min to enable proper CSF diffusion into the whole hippocampus. The CSF samples were obtained from four anti-NMDAR encephalitis patients and three epilepsy patients with confirmed absence of NMDAR-antibodies (hereafter referred to as control-CSF, Table 1). In addition, we injected artificial CSF (ACSF, composition see below) as a further control subgroup. The NMDAR-antibody titer was determined by end-point-titration of the characteristic NMDAR-antibody staining pattern on rat brain through indirect immunohistochemistry done by CB (Niehusmann et al., 2009); the NMDAR-antibody reactivity was confirmed by a cell-based assay performed by Angela Vincent (Oxford/UK) (Irani et al., 2010). After surgery, rats received metamizole (100–150 mg/kg) for postoperative pain control and allowed to recover in an enhanced O_2_ atmosphere (4–5 l/min in an 8-l glass vessel).

**Table 1:** Cerebrospinal fluid (CSF) samples.

CSF	Disease	NMDAR-ab titer	Age	Sex	# of animals
ACSF	n.a.	n.a.	n.a.	n.a.	4
Control-CSF C1	Epilepsy, focal cortical dysplasia	Negative	32	F	13
Control-CSF C2	Posttraumatic epilepsy	Negative	74	M	4
Control-CSF C3	Epilepsy, amygdala tumor	Negative	42	F	9
NMDAR-CSF N1	Epilepsy, anti-NMDAR encephalitis	1:32	20	M	8
NMDAR-CSF N2	Epilepsy, anti-NMDAR encephalitis	1:32	19	F	7
NMDAR-CSF N3	Epilepsy, anti-NMDAR encephalitis	1:512	26	F	7
NMDAR-CSF N4	Epilepsy, anti-NMDAR encephalitis	1:32	25	F	4

All patients gave their written informed consent to use their CSF samples for scientific purposes. The animal procedures were performed according to national and international guidelines on the ethical use of experimental animals (European Council Directive 86/609/EEC, approval of local authority LALLF M-V/TSD/7221.3-1.1-017/11 and LALLF M-V/TSD/7221.3-1.1-007/16); all efforts were made to minimize animal suffering and to reduce the number of animals used.

### Electrophysiological Recordings and LTP Induction

Hippocampal slices were prepared 1–8 days after stereotactic surgery as described previously (Würdemann et al., 2016), and the post-operative day was matched (control-CSF: 2.64 ± 0.34 days, *n* = 28; NMDAR-CSF: 2.65 ± 0.42 days, *n* = 26). Briefly, rats were decapitated in deep anesthesia with diethyl ether, the brains were rapidly removed and submerged into oxygenated ice-cold dissection solution containing (in mM) 125 NaCl, 26 NaHCO_3_, 3 KCl, 1.25 NaH_2_PO_4_, 0.2 CaCl_2_, 5 MgCl_2_ and 13 D-glucose (95% O_2_, 5% CO_2_; pH 7.4; 306–314 mosmol/kg). Then, 400 μm horizontal hippocampal brain slices were prepared using a vibratome (Campden Instruments, Loughborough, UK), and stored in a holding chamber containing ACSF containing (in mM) 125 NaCl, 26 NaHCO_3_, 3 KCl, 1.25 NaH_2_PO_4_, 2.5 CaCl_2_, 1.3 MgCl_2_ and 13 D-glucose (306–314 mosmol/kg, bubbled with 95% O_2_ and 5% CO_2_ to maintain the pH at 7.4).

For electrophysiological recordings, slices were transferred into a Haas type interface chamber and allowed to recover at least 30 min before recordings started. In the interface chamber, slices were continuously bathed in oxygenated ACSF (flow rate of 2 ml/min, temperature 32 ± 1°C, npi electronic GmbH, Tamm, Germany). Field excitatory postsynaptic potentials (fEPSP) were recorded in the CA3 “s.r.” (in Figure 1B_1_) for associational/commissural (A/C) fiber-evoked fEPSPs and in the CA3 “s.l.” (in Figure 1B_1_) for MF-evoked fEPSPs. In order to stimulate the afferent fibers, bipolar stimulating electrodes were fabricated from teflon-insulated platinum wire electrodes (PT-2T, Science Products, Hofheim, Germany) and placed within the CA3 s.r. (at the border between CA3 and CA2) for A/C fiber stimulation or within the CA3 s.l. (between the recording electrode and the dentate gyrus) for MF stimulation (Figure 1B_1_). The stimuli were delivered every 30 s through a stimulus isolator (A365, World Precision Instruments, Sarasota, FL, USA) triggered by a Master-8 stimulator (A.M.P.I., Jerusalem, Israel). At the beginning of the experiment, paired-pulse stimuli were delivered in order to document typical paired-pulse facilitation at the A/C fiber-CA3 synapse (fEPSP_2_-amplitude/fEPSP_1_-amplitude = 1.2–1.6; Figure 1B_2_). At the MF-CA3 synapse, the paired-pulse ratio, the characteristic 1 Hz frequency facilitation and the sensitivity to the metabotropic glutamate receptor agonist (2S,1’R,2’R,3’R)-2-(2,3-dicarboxycyclopropyl)glycine (DCG-IV, 3 μM) were used to confirm MF stimulation (Yoshino et al., 1996; Yeckel et al., 1999; Dietrich et al., 2003; Kirschstein et al., 2004).

Baseline stimulation intensity was adjusted to yield 40%–50% of the maximal fEPSP. The LTP induction protocol for A/C fiber-CA3 LTP consisted of 10 trains of 20 pulses at 100 Hz (stimulus duration 100 μs, intertrain interval 800 ms, referred to as modified delta burst stimulation, mdBS) at double baseline stimulation intensity. We used this protocol since these synapses express NMDAR-dependent LTP (Zalutsky and Nicoll, 1990) which is typically induced by burst stimulation paradigms (Grover et al., 2009). MF-LTP was induced by four tetanic trains of 100 pulses at 100 Hz (stimulus duration 100 μs, intertrain interval 3 s) at double baseline stimulation intensity, since tetanic protocols are commonly used to induce LTP at these synapses (Zalutsky and Nicoll, 1990; Dietrich et al., 2003; Schmitz et al., 2003). In all experiments with MF stimulation, the NMDAR antagonist D-2-amino-5-phosphonovalerate (D-AP5, 50 μM) was added. The analog recording data were amplified, filtered at 1 kHz by an EXT-10-2F (npi electronic GmbH, Tamm, Germany), and digitized using a Micro1401 analog-to-digital converter (Cambridge Electronic Design, Cambridge, UK) run by the Signal 2.16 software (Cambridge Electronic Design, Cambridge, UK). DCG-IV and D-AP5 were obtained from Tocris (Bristol, UK). All other chemicals used for physiological solutions were purchased from Sigma-Aldrich (Taufkirchen, Germany).

### Field Potential Analysis

Stimulation of A/C fibers evoked a field potential in CA3 s.r. that consisted of one or more peaks. The first peak was referred to as the fEPSP, and the maximal negative slope of this fEPSP was taken for the subsequent statistical analyses. In LTP experiments, all fEPSP slopes were calculated as the percentage of the fEPSP slope during the baseline phase. LTP was quantified by averaging the fEPSP slopes of the last 5 min of the experiment expressed as the percentage of the averaged baseline fEPSP slope. In case the first peak was followed by further peaks, these were referred to as epileptiform afterpotentials and counted. In order to allow an unbiased afterpotential count, the investigators (RB, WB, XG, TK) judged the number of afterpotentials without knowing the animal group. Afterpotentials were identified as local minima following the fEPSP that exceeded double peak-to-peak noise in 10 sweeps before mdBS (i.e., from −5 min to −0.5 min) and 10 sweeps after mdBS (i.e., from 0.5 min to 5 min). Then, we calculated the average number of epileptiform afterpotentials before and after the mdBS paradigm of LTP induction. In order to correlate epileptiform afterpotentials and LTP magnitudes, we calculated the fold change of the number of fEPSP peaks after mdBS to the number of fEPSP peaks under baseline conditions. To this end, we counted the number of fEPSP peaks (i.e., number of afterpotentials +1) before and after mdBS and then calculated the ratio of these numbers.

### Statistical Analysis

Data are expressed as mean values ± the standard error of the mean (SEM). All data were tested for normal distribution (SigmaStat 3.5) and then evaluated using the appropriate statistical test as indicated. The level of significance was set to *P* < 0.05; and significant differences were indicated with the exact *P* values when applicable.

## Results

### Associational-Commissural Fiber LTP in CA3 Is Impaired in Anti-NMDAR Tissue

**Figure 2:**
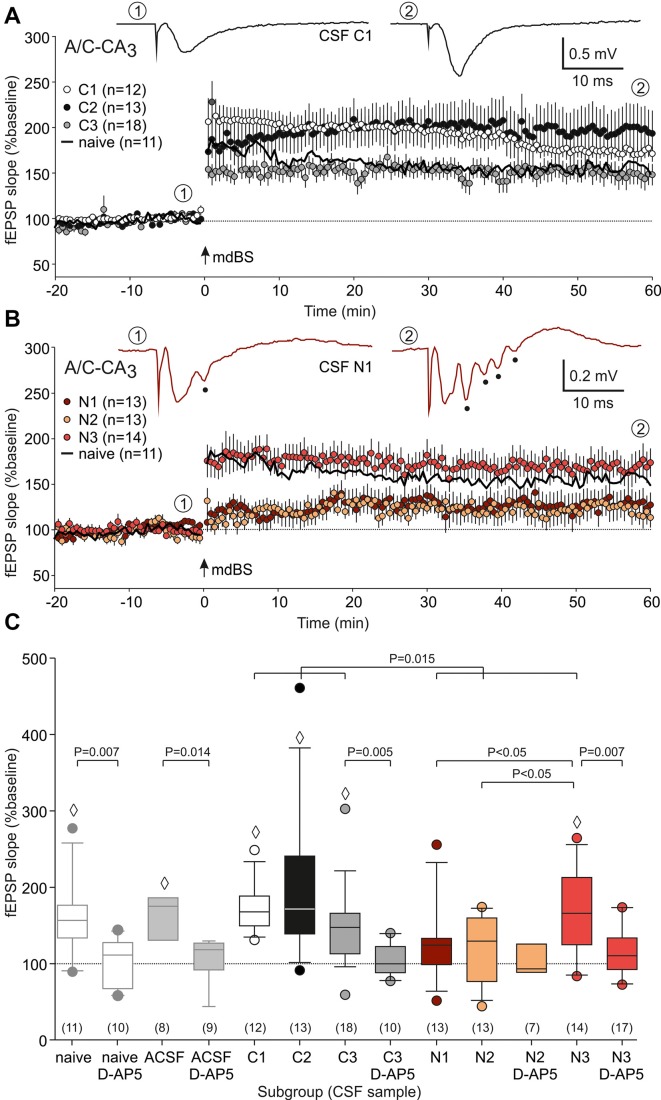
A/C fiber-CA3 long-term potentiation (LTP). **(A)** Time course of the mean relative field excitatory postsynaptic potential (fEPSP) slopes (expressed as percentage of the baseline fEPSP slope) of slices from control-CSF-treated animals. Note that robust LTP was obtained in all three subgroups tested (C1–C3). In addition, LTP from naive non-operated rats is indicated as a black line. The representative sample traces were taken from the subgroup C1 at the timepoints ① (baseline) and ② (end of the experiment). Modified delta burst stimulation (mdBS) was applied at timepoint “0” (indicated by an arrow). **(B)** Time course of the mean relative fEPSP slopes of slices from NMDAR-CSF-treated animals. Note that subgroups N1 and N2 only showed little residual potentiation, while the LTP magnitude of subgroup N3 was indistinguishable from control tissues. For the sake of clarity, LTP from naive non-operated rats is again indicated as a black line. The representative sample traces were taken from the subgroup N1 at the timepoints ① and ②, and they also illustrate the epileptiform afterpotentials (indicated by full circles). **(C)** Box-whisker plots of the LTP magnitude (mean relative fEPSP slope during the last 5 min of the experiment). *P* values for unpaired comparisons (with vs. without D-AP5 or control vs. NMDAR) were calculated using the Mann-Whitney U-test. The multiple test for NMDAR subgroups was performed using ANOVA followed by Student-Newman-Keuls posthoc test. The diamonds (◊) indicate significance of LTP (Wilcoxon signed rank test). The circles colored like the box-whisker plots indicate outliers.

The present study was performed in order to test whether NMDAR-dependent and independent LTP is differentially altered in a model of anti-NMDAR encephalitis. To this end, we stereotactically injected cell-free CSF from patients with anti-NMDAR encephalitis or from epileptic patients with proven absence of autoantibodies against these receptors (Figure 1A). First, we analyzed the NMDAR-dependent LTP at the A/C fiber input to CA3 pyramidal cells (Figure 1B_1_). A/C-CA3 responses were identified by the typical fEPSP shape and the characteristic paired-pulse ratio (Figure 1B_2_). We found that input-output (I/O) curves and the paired-pulse ratios (PPR) were not different between slices from control-CSF-injected and NMDAR-CSF-injected animals (I/O curve: *P* = 0.062, two-way-ANOVA; PPR: 0.315, Mann-Whitney U-test; Figures 1C,D). Hence, the presynaptic transmitter release was not affected by NMDAR-CSF injection and NMDAR-mediated components of the postsynaptic responses were largely intact. We then stimulated A/C fiber input in order to induce LTP. In naive non-operated animals, mdBS induced robust LTP at these synapses (157 ± 14% of baseline, *n* = 11; Figures 2A–C). Similar values of LTP at A/C fiber synapses were obtained when animals following intrahippocampal stereotactic injection with ACSF or cell-free control-CSF samples (C1–C3, Table 1) were used. Although the LTP magnitudes differed among the four control CSF subgroups (ACSF: 167 ± 13%, *n* = 8; C1: 173 ± 9%, *n* = 12; C2: 198 ± 25%, *n* = 13; C3: 148 ± 12%, *n* = 18; Figures 2A,C), there was no significant difference among all five control subgroups (H = 6.452 with df = 4, *P* = 0.168; Kruskal-Wallis H-test). Hence, under control conditions, A/C fiber LTP was on average 167 ± 8% (*n* = 62). NMDAR-dependance of A/C fiber LTP was confirmed using the NMDAR-inhibitor D-AP5 in naive (103 ± 9%, *n* = 10; *P* = 0.007 vs. naive without D-AP5, Mann-Whitney U-test), ACSF-injected (107 ± 9%, *n* = 9; *P* = 0.014 vs. ACSF without D-AP5, Mann-Whitney U-test) and control-CSF-injected animals (C3 + D-AP5: 104 ± 6%, *n* = 10; *P* = 0.005 vs. C3 without D-AP5, Mann-Whitney U-test; Figure 2C).

Next, we repeated these experiments using animals following stereotactic injection of CSF from anti-NMDAR encephalitis patients (N1–N3, Table 1). Here, we found again that the LTP magnitude differed substantially between the three NMDAR-CSF subgroups (N1: 127 ± 14%, *n* = 13; N2: 119 ± 12%, *n* = 13; N3: 168 ± 15%, *n* = 14; Figures 2B,C), but now reaching statistical significance (both N3 vs. N1 and N3 vs. N2, *P* < 0.05; ANOVA with Student-Newman-Keuls posthoc test; Figure 2C). This is an intriguing finding since animals treated with the highest NMDAR-antibody titer CSF (N3, Table 1) showed an LTP magnitude that was indistinguishable from controls. Nonetheless, overall A/C fiber LTP in NMDAR-CSF-treated animals (139 ± 9%, *n* = 40) was significantly reduced as compared to the averaged control LTP (*P* = 0.015; Mann-Whitney U-test; Figure 2C) indicating that intrahippocampal injection of CSF containing NMDAR-antibodies blocked NMDAR-dependent LTP at A/C fiber synapses. We were concerned about the observed variability of LTP measures among the different CSF subgroups. Since we matched the postoperative day in both control and anti-NMDAR groups, the postoperative delay was almost identical in both groups. In order to test for cohort effects that could have affected a particular subgroup, we correlated the post-operative day with the LTP magnitude in all experimental subgroups but found no correlation at all (control subgroups: *r* = −0.04, NMDAR-CSF subgroups: *r* = −0.06, Supplementary Figure S1).

Then, we tested the NMDAR inhibitor D-AP5 on LTP recorded in NMDAR-CSF-treated animals. As expected, the residual potentiation in slices from the N2 subgroup without NMDAR inhibition was not significantly different from the percentage change of the fEPSP slope in this subgroup with D-AP5 application (N2 + D-AP5: 103 ± 11%, *n* = 7; Figure 2C) indicating an almost complete LTP inhibition by CSF containing NMDAR-antibodies. On the other hand, the significant potentiation observed in the N3 subgroup was significantly reduced by NMDAR inhibition and hence no longer statistically significant when compared to baseline (N3 + D-AP5: 116 ± 7%, *n* = 17; *P* = 0.007 vs. N3, Mann-Whitney U-test; *P* = 0.051 vs. baseline, Wilcoxon signed rank test; Figure 2C). These data suggest that the NMDAR-antibody from this patient did not block NMDAR-dependent LTP at the A/C fiber-CA3 synapse.

### Epileptiform Afterpotentials Are More Common in Anti-NMDAR Tissue

**Figure 3:**
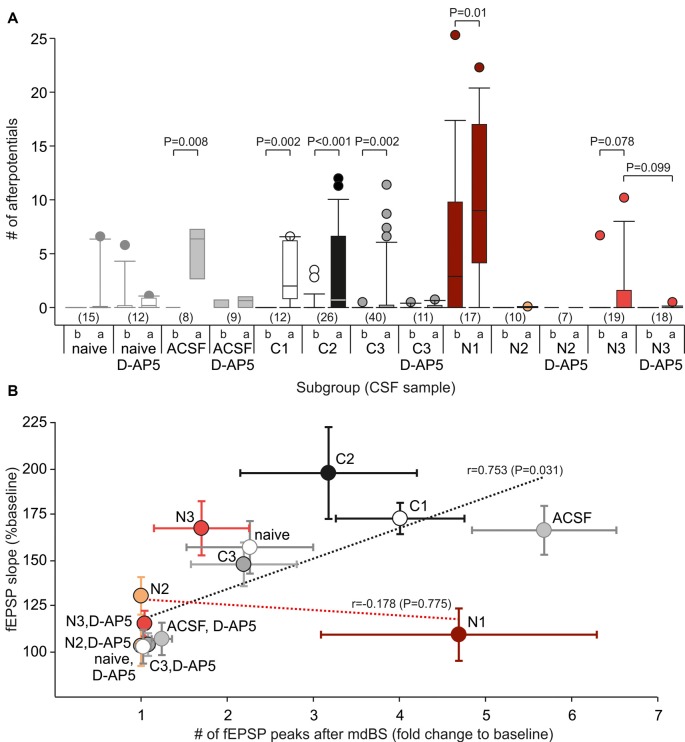
Epileptiform afterpotentials are associated with A/C fiber LTP. **(A)** Box-whisker plots of the number of afterpotentials following the primary negative deflection referred to as the fEPSP for all subgroups (see Table 1 for CSF data). The lowercase letters indicate that the number of afterpotentials was counted before (“b”) or after (“a”) application of the mdBS paradigm. *P* values for paired comparisons (before vs. after mdBS) were obtained by using the Wilcoxon rank sum test, *P* values for unpaired comparisons (with vs. without D-AP5) were obtained by using the Mann-Whitney U-test. Circles colored like the box-whisker plots indicate outliers. **(B)** Correlation between the number of afterpotentials and the LTP magnitude (fEPSP slope as percentage of baseline value) for all subgroups. There was a positive correlation for control-CSF-treated subgroups, but not for NMDAR-CSF-treated tissue (Pearson correlation coefficient, *t*-test).

The data so far point to a deficit in NMDAR-dependent LTP at A/C fibers in the hippocampal subfield CA3 in animals injected with CSF containing NMDAR antibodies. As shown by the sample traces in Figure 2B, the fEPSPs in CA3 occasionally showed epileptiform afterpotentials following the fEPSP (indicated by points below the trace), especially after the mdBS protocol. We calculated this systematically by counting the mean number of afterpotentials for the last 5 min before mdBS (referred to as “b” in Figure 3A) as well as for the first 5 min after mdBS (referred to as “a” in Figure 3A). Hence, epileptiform afterpotentials were never seen in slices from naive, non-operated animals before mdBS, and only rarely present after mdBS (3/15 slices, Figure 3A). In slices from control-CSF-injected animals, epileptiform afterpotentials were also extremely rare before mdBS (ACSF: 1/8 slices; C1–C3: 4/78 slices). However, in all control-CSF subgroups, we observed a significant increase of epileptiform afterpotentials following mdBS (in 43/86 slices, *P* < 0.001, *χ*^2^ test; Figure 3A). With respect to the NMDAR-CSF-injected subgroups, we observed a higher variance in the presence of epileptiform afterpotentials. In particular, 11 of 17 slices from the N1 subgroup showed afterpotentials before mdBS (5.9 ± 1.7, *n* = 17). Following mdBS, however, afterpotentials were seen in all but two slices, and the number of afterpotentials increased significantly (9.8 ± 1.7, *n* = 17; *P* = 0.01 vs. pre-mdBS, Wilcoxon signed rank test). In contrast, slices from the NMDAR-CSF-injected subgroup N2 never showed epileptiform afterpotentials (Figure 3A). In addition, slices from the high-titer N3 subgroup rarely showed afterpotentials before mdBS (1/19 slices), which were rather common after mdBS (7/19 slices; *P* = 0.042, Fisher’s exact test), although the increased number of afterpotentials did not reach statistical significance (*P* = 0.078, Wilcoxon signed rank test).

Next, we tested whether epileptiform afterpotentials were sensitive to the NMDAR inhibitor D-AP5. As it can be appreciated from Figure 3A, adding D-AP5 to the bath solution seemed to block epileptiform afterpotentials. In order to assess this in more detail, we counted the slices with presence or absence of epileptiform afterpotentials and performed contingency table analyses (Table 2). Under control-CSF conditions, the proportion of slices showing epileptiform afterpotentials was not significantly reduced by D-AP5. Moreover, when all control slices were pooled together, D-AP5 rather tended to enhance the proportion of slices showing epileptiform afterpotentials (without D-AP5: 5/101 slices, with D-AP5: 7/32 slices, *P* = 0.011, *χ*^2^ test). On the contrary, D-AP5 almost prevented epileptiform afterpotentials in NMDAR-CSF-treated tissue (before mdBS: 0/25, *P* = 0.006, Fisher’s exact test; after mdBS: 3/25, *P* = 0.002, Fisher’s exact test; Table 2). Hence, D-AP5 specifically inhibited the appearance of epileptiform afterpotentials in NMDAR-CSF-treated tissue, but not in control tissue.

**Table 2:** Contingency tables of epileptiform afterpotentials.

	Without D-AP5		With D-AP5		*P* value (D-AP5 effect)
Epileptiform afterpotentials	Present	Absent	Present	Absent	
ACSF, before mdBS	1 (13%)	7 (87%)	2 (22%)	7 (78%)	1.000
ACSF, after mdBS	8 (100%)	0 (0%)	6 (67%)	3 (33%)	0.206
Control-CSF, before mdBS	4 (5%)	74 (95%)	1 (9%)	10 (91%)	0.491
Control-CSF, after mdBS	35 (45%)	43 (55%)	3 (27%)	8 (73%)	0.341
NMDAR-CSF, before mdBS	12 (26%)	34 (74%)	0 (0%)	25 (100%)	0.006*
NMDAR-CSF, after mdBS	23 (50%)	23 (50%)	3 (12%)	22 (88%)	0.002*

*The data indicate the number of slices (and proportions) in bath solution without D-AP5 or with D-AP5 showing epileptiform afterpotentials (present) or not (absent) in ACSF, control-CSF and NMDAR-CSF-treated animals. The P value for the D-AP5 effect was calculated using the Fisher’s exact test. Significant P values are indicated by asterisks*.

In summary, under baseline conditions, epileptiform afterpotentials were present in 5 of 86 slices from ACSF and control-CSF-treated animals, but in 12 of 46 slices from NMDAR-CSF-treated animals (*P* = 0.002, *χ*^2^ test). The LTP-inducing mdBS paradigm facilitated the occurrence of afterpotentials following the first fEPSP peak in both control and anti-NMDAR subgroups. However, the NMDAR blocker significantly reduced the proportion of slices with epileptiform afterpotentials only in tissue from NMDAR-CSF-treated animals, but not in control tissue. These findings indicate that the stereotactic injection of anti-NMDAR CSF exerts a facilitative effect on the presence of epileptiform afterpotentials and suggest that this facilitation is mediated by targeting NMDA receptors.

Since we found substantial subgroup differences in the LTP magnitude as well as in the number of epileptiform afterpotentials after mdBS application, we aimed to correlate these measures. Therefore, we calculated the fold change of the number of fEPSP peaks after mdBS to the number at baseline conditions and found a high correlation between this change and LTP in control subgroups (*r* = 0.753, *P* = 0.031, *t*-test; Figure 3B). Thus, in control-CSF-treated tissue, LTP facilitation was accompanied by some hyperexcitability as assessed by the number of afterpotentials. In contrast to controls, NMDAR-CSF-treated tissue did not show any correlation between LTP achieved and the number of afterpotentials (*r* = 0.178, *P* = 0.775, *t*-test; Figure 3B). This finding points to some variation of the NMDAR-autoantibody epitope among different patients and, in addition, suggests that different NMDAR-dependent processes, such as LTP and epileptiform afterpotentials, may involve differently composed NMDAR complexes.

### Mossy Fiber LTP in CA3 Is Intact in Anti-NMDAR Tissue

**Figure 4:**
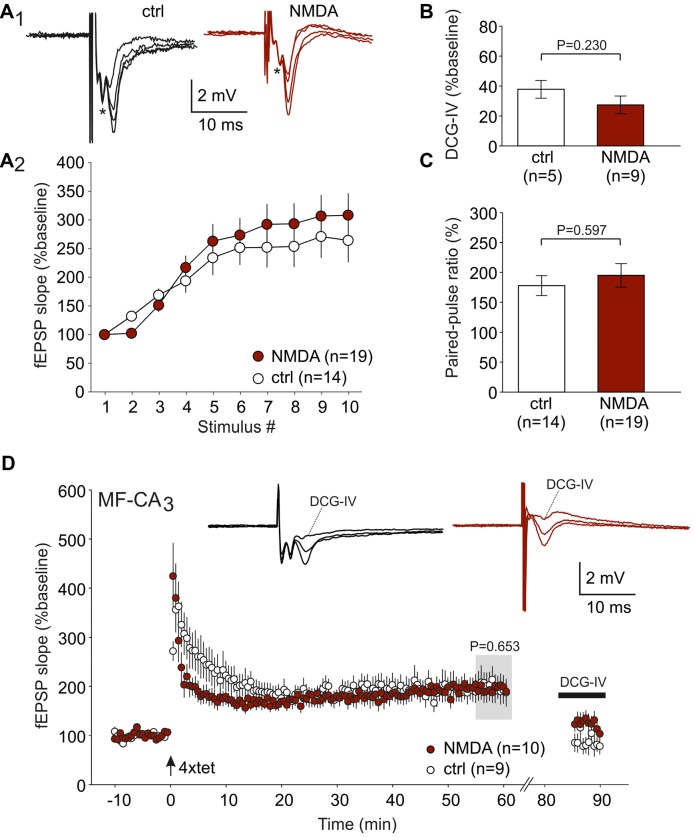
MF LTP is intact in anti-NMDAR tissue. **(A)** Frequency facilitation (1 Hz) shows no significant difference between NMDAR-CSF-treated and control-CSF-treated tissue. **(A1)** Examples of original recordings. **(A2)** Summary plot. The asterisk (*) in panel **(A_1_)** indicates the presynaptic fiber volley which remained constant during the 1 Hz stimulation paradigm. **(B)** Residual fEPSP amplitude following DCG-IV (expressed as the percentage of the baseline response). Note that the sensitivity towards the group II metabotropic glutamate receptor agonist DCG-IV was not different between control and anti-NMDAR tissue (*P* value calculated by using Mann-Whitney U-test). **(C)** The paired-pulse ratio did not significantly differ between both experimental groups (*P* value calculated by using Mann-Whitney U-test). **(D)** LTP at the MF synapse showed almost equal magnitudes at the end of the experiment for both NMDAR-CSF- and control-CSF-treated tissue. Note that DCG-IV significantly reduced the potentiated fEPSPs in both groups. Insets show typical recordings before and after tetanic stimulation as well as after DCG-IV.

Since MF fiber LTP is NMDAR-independent (Harris and Cotman, 1986; Williams and Johnston, 1988), we took the opportunity to study this form of LTP in animals following NMDAR-CSF-treatment (CSF N1 and N4). In order to avoid contamination of A/C fiber evoked potentiation, we added D-AP5 in all experiments. First, we tested typical short-term plasticity paradigms for MF synapses. Upon 1 Hz stimulation, significant facilitation was observed in both control and NMDAR-CSF-treated animals (264 ± 34%, *n* = 14 and 308 ± 38%, *n* = 19, respectively; Figure 4A1,A2). The two-way ANOVA detected significant stimulation effect (*P* < 0.001), but there was no significant animal group effect (*P* = 0.156; Figure 4A2). Sensitivity to the group II metabotropic glutamate receptor agonist DCG-IV is a key finding of MF-evoked fEPSPs (Yoshino et al., 1996; Yeckel et al., 1999; Dietrich et al., 2003). However, the residual fEPSP following DCG-IV treatment was also similar in both groups (control: 38 ± 6%, *n* = 5; anti-NMDAR: 27 ± 6%, *n* = 9; *P* = 0.230, Mann-Whitney U-test; Figure 4B) confirming the MF input in both animal groups. The paired-pulse ratio with interstimulus interval of 40 ms was not significantly different either (control: 178 ± 17%, *n* = 14; anti-NMDAR: 195 ± 20%, *n* = 19; *P* = 0.597, Mann-Whitney U-test; Figure 4C). Thus, there were no significant differences between control-CSF and NMDAR-CSF-treated animals in these forms of short-term plasticity. Then, we performed LTP experiments using tetanic stimulation (four trains of 100 stimuli). As shown in Figure 4D, robust potentiation was induced at MF synapses in both control slices (C1: 199 ± 26%, *n* = 9) and slices from NMDAR-CSF-treated animals (195 ± 22%, *n* = 10; *P* = 0.653 vs. control, Mann-Whitney U-test; Figure 4D). Importantly, the LTP magnitudes were almost identical suggesting that CSF containing NMDAR-antibodies did not affect this form of LTP. At the end of the experiment, sensitivity to DCG-IV confirmed that LTP under these conditions was predominantly mediated by MF stimulation (Figure 4D).

## Discussion

### NMDAR Specificity of CSF From Anti-NMDAR Encephalitis

The present study was conducted to address the specificity of CSF from patients with anti-NMDAR encephalitis. To this end, we chose a hippocampal subfield where both NMDAR-dependent and NMDAR-independent forms of LTP are expressed in adjacently located synapses. In the CA3 region, the former is found at the A/C fiber synapse, the latter is expressed at the MF synapse. Our data demonstrate that, on average, A/C fiber LTP was significantly suppressed in slices from NMDAR-CSF-treated animals whereas MF LTP remained intact in these animals. Since A/C fiber LTP could be abolished by the NMDAR blocker D-AP5, but MF LTP was induced in the presence of NMDAR inhibition, our findings demonstrate that CSF from anti-NMDAR encephalitis patients selectively blocks NMDAR-dependent LTP but leaves NMDAR-independent LTP unaltered.

The finding that NMDAR-dependent LTP at the A/C fiber synapse is reduced by NMDAR-CSF is consistent with a series of recent reports that have also demonstrated LTP impairment by both commercial and patient-derived NMDAR-antibodies (Zhang et al., 2012; Dupuis et al., 2014; Würdemann et al., 2016). More precisely, LTP impairment was shown at the perforant path-dentate gyrus synapse as well as at the Schaffer collateral-CA1 synapse, both of which express NMDAR-dependent LTP (Collingridge et al., 1983; Harris et al., 1984; Morris et al., 1986). Since the A/C fiber-CA3 synapse has not been studied so far with respect to its sensitivity towards anti-NMDAR, the present findings augment our current knowledge about these antibodies. In the view of the previous literature, our data suggest that the impaired NMDAR function at A/C fiber-CA3 synapses may be additive to the deficit in NMDAR function, very likely brought about by receptor internalization (Hughes et al., 2010) at the synapses studied so far and, hence, be co-involved in the cognitive phenotype of anti-NMDAR encephalitis patients.

While overall LTP at A/C fibers was depressed in tissue from anti-NMDAR-treated animals, the present study also revealed significant variation among the three NMDAR-CSF subgroups tested. Stereotactic application of CSF sample N3 failed to intervene with A/C fiber LTP, but interleaved experiments with D-AP5 also proved the NMDAR dependance of LTP in anti-NMDAR tissue from this subgroup. Although high CSF titers could generally be due to lack of antibody binding to the receptor, these data suggest that the presence of NMDAR-antibodies does not necessarily mean that all NMDARs are targets for these autoantibodies. On the molecular level, the epitope of NMDAR-antibodies has been clearly attributed to the N368/G369 residues of the GluN1 subunit (Kreye et al., 2016; Dalmau et al., 2017), and the GluN1 subunit was convincingly demonstrated to be pathogenically relevant in a passive transfer model from man to mouse (Malviya et al., 2017). Hence, it is intriguing to speculate whether different epitope accessibility within the tissue might explain some variation. Certainly, there are methodological issues that may cause some variability. First, the site of injection, although verified by interleaved ink applications, carries some intrinsic variation and, hence, misplaced injections cannot fully be ruled out. Second, diffusion and probably consumption of the NMDAR-antibodies take place during the postoperative period and will lead to some variation as well. However, our previous study demonstrated that behavioral effects were visible as late as 14 days after surgery (Würdemann et al., 2016) indicating that diffusion within the brain parenchyma is a rather slow process. Third, interspecies problems when injecting human CSF into rodent hippocampus may also be an issue.

But besides methodological aspects, an important issue may also be that patient-derived CSF samples may contain not yet known pathogenic components in addition to NMDAR-antibodies such as autoantibodies against other targets. In addition, treatment with EphrinB2, the ligand of the EphB2 receptor, reversed the effects of patient-derived NMDAR-antibodies (Planagumà et al., 2016). These intriguing results may indicate that endogenous factors may also play a major role in fine-tuning NMDAR-antibody effects and intrinsic interaction partners of NMDAR surface expression might thus become promising strategies beyond immunotherapy. With respect to our study, it is conceivable that endogenous factors in the CSF from patients affect the NMDAR-antibody binding to its targets in the rat brain parenchyma. Both additional pathogenic agents and endogenous factors cannot be ruled out and may significantly add to variation.

What is known about NMDAR subunit expression in CA3? Recently, the CA3 pyramidal neuron was found to express significantly more GluN2B at the A/C fiber synapse as compared to the MF synapse (Carta et al., 2018). Thus, a single neuron can specifically direct NMDAR complexes and possibly also differently composed NMDAR complexes to certain synapses. It is possible that this could be a key molecular mechanism of central neurons enabling specific postsynaptic responses to transmitter release from certain presynaptic terminals, referred to as synapse input specificity. It is important to note that fibers from the entorhinal cortex also terminate directly in the CA3 area which may additionally be recruited by the LTP-inducing stimulation. It has been shown that direct perforant path-CA3 synapses and A/C fiber-CA3 synapses exhibit associative LTP (Martinez et al., 2002), i.e., weak activity at one pathway can be compensated for by activity of the other in order to produce a common form of postsynaptic LTP. In addition, perforant path-CA3 LTP is NMDAR-dependent (McMahon and Barrionuevo, 2002), and we cannot exclude that NMDARs at perforant path-CA3 synapses had been reached by NMDAR-antibodies in our model and contributed to A/C fiber LTP.

### Epileptogenicity of CSF From Anti-NMDAR Encephalitis

Clinically, seizures are very common in anti-NMDAR encephalitis, but it is difficult to explain the epileptic condition by the commonly proposed mechanism of NMDAR hypofunction due to receptor cross-linking and internalization (Lynch et al., 2018). It has been shown that NMDAR-antibodies may directly impact the NMDAR gating behavior leading to more frequent channel openings and prolonged open times of the receptor (Gleichman et al., 2012). This could at least be relevant in slices from the N1 subgroup presenting with excessive epileptiform afterpotentials.

One attractive, but unresolved explanation for epileptic activity by NMDAR hypofunction is the action of NMDAR-antibodies at NMDARs on GABAergic interneurons, very likely within the hippocampus. In previous reports, either the epileptic phenotype has not been addressed (Dupuis et al., 2014) or CSF from anti-NMDAR-encephalitis patients could not evoke or even facilitate epileptiform activity (Planagumà et al., 2015; Würdemann et al., 2016). The reasons for this are not well understood, but one potential issue might be strain-dependent sensitivity to epilepsy-inducing strategies (Löscher et al., 1998; Ferraro et al., 2002). In the present study, however, we found that epileptiform afterpotentials following the A/C fiber-stimulation evoked fEPSP were significantly more frequent in slices from NMDAR-CSF-treated animals as compared to control-CSF-treated tissue. Importantly, epileptiform potentials were not observed in naive, non-operated animals indicating a specific effect after stereotactic CSF injection. Epileptiform afterpotentials were not detected in our previous study on the perforant path-dentate gyrus synapse (Würdemann et al., 2016), but at the Schaffer collateral-CA1 synapse, one previous study even showed fEPSP sample traces with additional components that could represent epileptiform afterpotentials although not quantitatively analyzed in this study (Zhang et al., 2012). Hence, our data are consistent with the idea that NMDAR-antibodies might not only address pyramidal cell-expressing NMDARs, but also NMDARs on GABAergic interneurons, most probably located within the hippocampal subfield CA3.

The CA3 interneuronal network has attracted less attention in the past, but in recent years, this network has come into the focus of several CA3 plasticity studies. On the one hand, the CA3 network is highly divergent and one CA3 pyramidal cell may even drive several CA3 interneuron classes. For instance, CA3 pyramidal cells were found to innervate parvalbumin-positive basket cells more extensively than axo-axonic cells, but the NMDAR-mediated EPSC component had been similar in both types of interneurons (Papp et al., 2013). On the other hand, the CA3 network is also highly convergent. Stratum radiatum/stratum lacunosum-moleculare interneurons receive input from both CA3 pyramidal cells via recurrent A/C fibers and dentate gyrus granule cells via MFs. Importantly, A/C fiber LTP, but not MF LTP on these interneurons was CaMKII-dependent, and, in turn, protein kinase A was involved in MF LTP, but not A/C fiber LTP (Galván et al., 2015). With respect to the preferred interaction between CaMKII and GluN2B, it is an intriguing question whether different NMDAR subunit compositions might also be involved in the input specificity of these interneurons.

NMDAR hypofunction of GABAergic interneurons should result in a disinhibited state of the CA3 network and thus displays an attractive mechanism for epileptogenesis in anti-NMDAR encephalitis. CA3 has recently been found to be the seizure onset zone in most animals following pilocarpine-induced status epilepticus (Behr et al., 2017; Samiee et al., 2018) suggesting that CA3 is a particular epilepsy-prone area. Based on these data and our observation that the LTP-inducing stimulation even facilitated the occurrence of epileptiform afterpotentials, we suggest that NMDAR-CSF perturbs the balance between NMDAR-mediated transmission at A/C fiber-CA3 synapses and NMDAR-mediated drive from CA3 pyramids, dentate granule cells and possibly entorhinal cortices onto CA3 interneurons.

The LTP induction protocol significantly increased the prevalence of epileptiform afterpotentials in slices from both control-CSF- and NMDAR-CSF-treated animals, but also, albeit very rarely, in slices from naive rats. In most cases, however, only one additional component was obtained, and it is very likely that this reflected the fEPSP decay phase after a population spike rather than any epileptiform afterpotential. Nonetheless, even though we formally counted these components as epileptiform afterpotentials, it is of note that the NMDAR blocker D-AP5 lowered the prevalence of such components only in NMDAR-CSF-treated, but not in control-CSF-treated tissue. Intriguingly, this was also the case for the NMDAR-CSF subgroup N3 which did not block A/C fiber LTP at all. In summary, D-AP5 blocked both epileptiform afterpotentials and LTP. An unexpected finding, however, was that D-AP5 rather tended to raise the proportion of slices showing epileptiform afterpotentials. This might indicate that the most parsimonious explanation—simply diminishing the excitability by acting on remaining NMDARs following D-AP5 application—may not fully explain this unexpected finding. Therefore, the NMDAR specificity of the enhanced incidence of epileptiform potential components associated with LTP-inducing stimuli and may suggest that epitope accessibility of pyramidal cell-expressed and interneuron-expressed NMDAR complexes might be an issue in future.

In summary, our data presented here showed that CSF containing NMDAR-antibodies impaired LTP at the A/C fiber-CA3 synapse, but left MF LTP unaltered. This demonstrates NMDAR specificity of patient-derived CSF, but beyond this, differential suppression of A/C fiber LTP and the newly-discovered facilitation of epileptiform activity suggest that autoantibodies may differentiate certain NMDAR subunit compositions and thereby excitatory and inhibitory neurons in CA3.

## Author Contributions

RB, WB, XG, KP and TS performed experiments. CB, RK and TK contributed to the conception and design of the study. RB, WB and XG organized the database and contributed to manuscript preparation. RB, WB, XG and TK performed the statistical analysis. TK wrote the first draft of the manuscript. CB and RK wrote sections of the manuscript. All authors contributed to manuscript revision, read and approved the final version of this manuscript for submission.

## Conflict of Interest Statement

The authors declare that the research was conducted in the absence of any commercial or financial relationships that could be construed as a potential conflict of interest.

## Supplementary Material

FIGURE S1 Correlation between the postoperative day and the long-term potentiation (LTP) magnitude for all subgroups. There is no significant correlation between postoperative day and LTP magnitude (field excitatory postsynaptic potentials (fEPSP) slope as percentage of baseline value), neither among control-CSF subgroups, nor among NMDA receptors (NMDAR)-CSF subgroups.
